# Commentary: Identification of optimal reference genes for gene expression normalization in human osteosarcoma cell lines under proliferative conditions

**DOI:** 10.3389/fgene.2024.1342447

**Published:** 2024-01-25

**Authors:** Paulo R. C. de Sousa, Andreon S. M. da Silva, Carlos G. G. de Ponte, Lucas L. Nogueira, Cristiane C. Frota, Paulo R. Z. Antas

**Affiliations:** ^1^ Programa de Pós-Graduação em Patologia, Universidade Federal do Ceará, Fortaleza, Brazil; ^2^ Fundação Oswaldo Cruz, Rio de Janeiro, Brazil

**Keywords:** real-time quantitative polymerase chain reaction, reference genes, 18S rRNA analysis, THP-1 cell line, *in silico* prediction software

**To the Editor—**We have read with interest the paper by Dong et al. (2022), an essay on identification of ideal reference genes (RGs) for expression analysis, which showed that using three algorithms plus an *in silico* prediction software application, the ribosomal RNA (18S rRNA) was among the genes with the most stable expression. Preceding that, Silveira et al. (2021) highlighted the significance of assessing the expression stability of RGs systematically when performing real-time quantitative polymerase chain reaction (qRT-PCR) analysis under different experimental conditions. Because qRT-PCR requires stable and reliable genes for normalization, it is crucial to select and validate, and to compare and reproduce meaningful analyses of expression changes of the genes of interest (Dundas and Ling, 2012).

**FIGURE 1:**
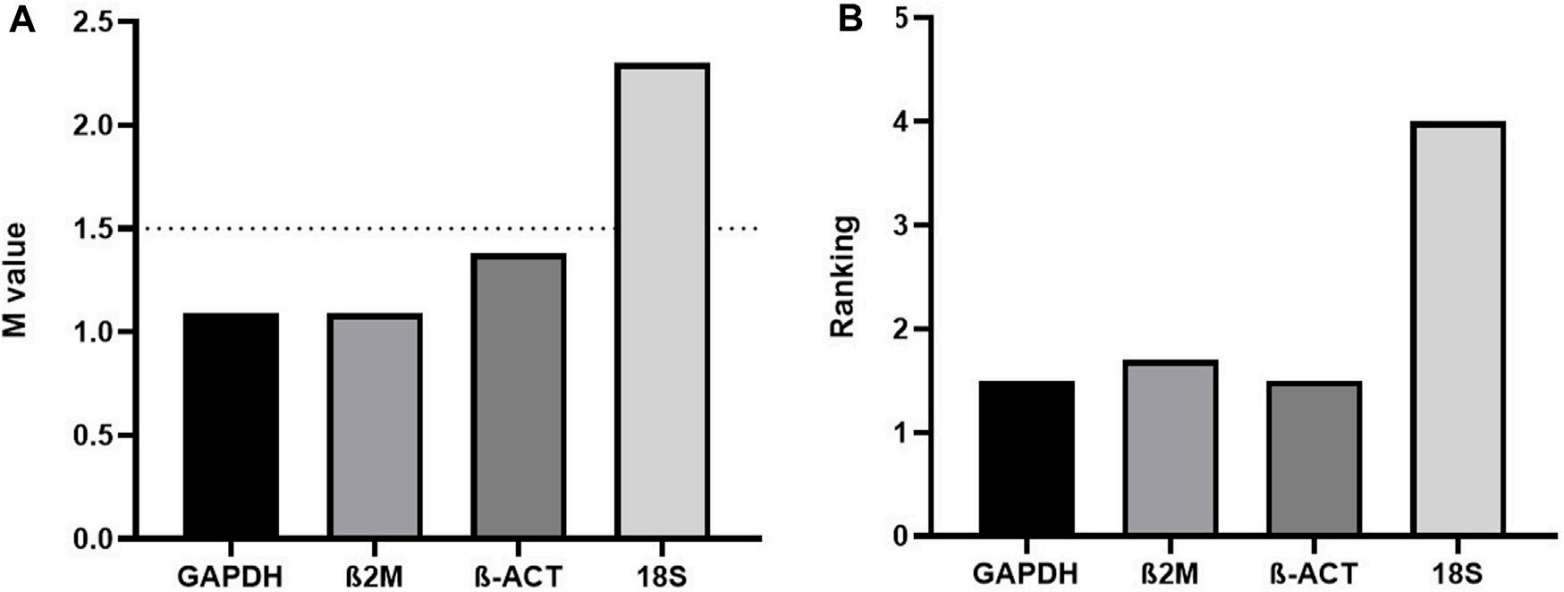
**(A)** geNorm and **(B)** RefFinder expression stability (M) and ranking values of four reference genes tested in the THP-1 cell line stimulated with Brefeldin A for 16 h (*n* = 13). β2M=β-2-microglobulin; β-ACT=β-actin.

Regarding this latter topic, we would like to comment on important fresh, restricted data. When assaying the human acute monocytic leukemia THP-1 cell line stimulated with 1 mM of Brefeldin A (Hypofarma, Brazil) for 16 h, we uncovered significant information concerning the determination of the expression stability, leading to the choice of the best housekeeping genes for gene expression analysis in a real-time qRT-PCR study. For this, we selected and concomitantly tested in duplicates the stability of four most commonly used RGs in immunological studies using the GoTaq qPCR Master Mix kit (Promega Biosciences, United States) in ABI 7500 Fast equipment (Applied Biosystem, United States): *GAPDH*, *β-actin*, *18S rRNA*, and *β-2-microglobulin* [(Dundas and Ling, 2012; Chapman and Waldenström, 2015)]. The details are given in Table 1. As determined previously [(Freitas et al., 2019; Vandesompele et al., 2002)], RG with a lower geNorm mean value of expression stability (M) was considered trustworthy. Hence, three candidate RGs, namely, β-actin, β-2-microglobulin, and GAPDH, displayed satisfactory results (M-values <1.5), except for the 18S rRNA gene, which was considered the least stable (M-value = 2.4) under our test conditions (Figure 1A). This finding replicated other studies [(Molomjamts and Ingolfsland, 2023; Lewczuk et al., 2023; Gubern et al., 2009; Julian et al., 2014; Zhang et al., 2018)]. Based on this, we realized that the 18S rRNA gene performed worst during human mRNA analysis *in vitro* upon drug stimulus due to its unstable expression. Concordantly, all three prior candidate RGs were again deemed to be potentially mostly stable by RefFinder, with ranking values very close to each other (Figure 1B). Once more, the 18S rRNA gene was considered poorly stable by that algorithm. Despite rRNA comprising roughly 80% of the total RNA available in the cell, being less prone to degradation, our data are in contrast with Dong et al. (2022) and two other controversial studies [(Song et al., 2022; Köhsler et al., 2020)]. Importantly, to ensure the specificity of the amplified products in real-time qRT-PCR assays, the fluorescence signals obtained after the melting curve analysis for each amplification cycle confirmed to yield unique and reproducible peaks in the different replicates analyzed (data not shown). In addition, the cDNA samples presented the parameters considered reliable for application in real-time qRT-PCR assays, ranging from 1.8 to 2.2 in efficiency values (data not shown).

**TABLE 1:** Description of target housekeeping genes and primers used in qRT-PCR assays.

Gene name *(GenBank accession no.)*	^#^DNA sequence 5’ → 3’	Amplicon (pb)	*E
**18S**	F:TGTGCCGCTAGAGGTGAAATT	63	1.87
*(XM_047442803.1)*	R:TGGCAAATGCTTTCGCTTT		
**GAPDH**	F:GGTGTGAACCATGAGAAGTATGA	123	1.91
*(NM_001357943.2)*	R:GAGTCCTTCCACGATACCAAAG		
**β-2-Microglobulin**	F:GCTCCGTGGCCTTAGCTGT	89	1.84
*(NM_004048.4)*	R:ACGTGAGTAAACCTGAATCTTTGGA		
**β-Actin**	F:ATTGCCGACAGGATGCAGAA	150	1.84
*(NM_001101.5)*	R:GCTGATCCACATCTGCTGGAA		

^**#**^The sequences were designed for the targets using the PrimerQuest Tool software (Intregrated DNA Technologies, United States).

*E, Efficiency calculated from linear regression analyses and slope values of the Ct’s of each dilution.

Identifying and validating optimal internal RNA standards for a real-time qRT-PCR essay, either in constitutive or stimulatory expression patterns under different conditions, is essential for the accurate and reproducible measurement of gene expression abundance in a specific cell or tissue (Yi et al., 2020; Bustin et al., 2010). Furthermore, it is critical to screen for stable endogenous control genes as there are no universally applicable RGs with invariant expression available; therefore, more than one RG should be employed in order to avoid such a bias in the interpretation of results (Kozera and Rapacz, 2013). Therefore, normalizing a qRT-PCR dataset to 18S rRNA may result in faulty data, at least when measuring mRNA levels from the THP-1 cell line model, whereas RG amplified products employing β-actin, GAPDH, and β-2-microglobulin mRNA might lead to more reliable datasets. GAPDH, a commonly used RG in several studies, has been found to be more suitable (Dundas and Ling, 2012). Alternatively, β-actin was the most appropriate RG to be used as an endogenous control for mRNA quantification in cell line studies (Bronkhorst et al., 2016).

In summary, it is crucial to carefully evaluate ahead for appropriate RG in expression studies for the accurate detection of meaningful changes in targeted gene transcript levels in human *in vitro* real-time qRT-PCR studies since normalization with unsuitable housekeeping gene products led to significant misinterpretation of expression profiles and final results (Köhsler et al., 2020). Our data not only support those previous ones (Molomjamts and Ingolfsland, 2023; Lewczuk et al., 2023; Gubern et al., 2009; Julian et al., 2014; Zhang et al., 2018) but also add another round of information regarding the selection and validation of ideal RGs for expression analysis. Finally, the ideal RG should have minimal regulation across the disease spectrum and be minimally influenced by patient heterogeneity (Kumar et al., 2022). Thus, other follow-up studies are warranted to better clarify this important issue.
